# Dendritic Cell Podosome Dynamics Does Not Depend on the F-actin Regulator SWAP-70

**DOI:** 10.1371/journal.pone.0060642

**Published:** 2013-03-27

**Authors:** Anne Götz, Rolf Jessberger

**Affiliations:** Faculty of Medicine Carl Gustav Carus, Institute of Physiological Chemistry, Dresden University of Technology, Dresden, Germany; University of Illinois at Chicago, United States of America

## Abstract

In addition to classical adhesion structures like filopodia or focal adhesions, dendritic cells similar to macrophages and osteoclasts assemble highly dynamic F-actin structures called podosomes. They are involved in cellular processes such as extracellular matrix degradation, bone resorption by osteoclasts, and trans-cellular diapedesis of lymphocytes. Besides adhesion and migration, podosomes enable dendritic cells to degrade connective tissue by matrix metalloproteinases. SWAP-70 interacts with RhoGTPases and F-actin and regulates migration of dendritic cells. SWAP-70 deficient osteoclasts are impaired in F-actin-ring formation and bone resorption. In the present study, we demonstrate that SWAP-70 is not required for podosome formation and F-actin turnover in dendritic cells. Furthermore, we found that toll-like receptor 4 ligand induced podosome disassembly and podosome-mediated matrix degradation is not affected by SWAP-70 in dendritic cells. Thus, podosome formation and function in dendritic cells is independent of SWAP-70.

## Introduction

Dendritic cells (DCs) are the most efficient antigen presenting cells and act as key regulators of the immune system [1]. Immature DCs reside in peripheral tissues and become activated either spontaneously or by encounter with pathogen molecules. Upon activation, DCs undergo a tightly regulated maturation process that enables them to migrate to secondary lymphoid organs to initiate immune responses. This maturation process is typically accompanied by changes in DC morphology and behavior. For example, DCs rearrange their actin cytoskeleton to migrate rapidly through complex environments in the interstitium. Activated DCs also up-regulate co-stimulatory molecules to induce either immunity or tolerance by presenting acquired antigens to naïve T cells in lymph nodes [1], [2]. Hence, elucidating the mechanisms that regulate DC migration from peripheral tissues to lymph nodes upon activation is essential to understand central functions of the immune system.

To facilitate migration through the extracellular matrix, DCs need to adhere to their environment with specific adhesion structures. In addition to classical adhesion structures like filopodia or focal adhesions, DCs and other cells like macrophages and osteoclasts assemble highly dynamic actin structures called podosomes [3]. These F-actin-based adhesion structures share many, but not all, proteins and are structurally and functionally different: the F-actin-core within podosomes is oriented perpendicular to the plasma membrane whereas focal adhesions display elongated F-actin structures with tangential orientation to the substrate. To facilitate movement focal adhesions are connected with the F-actin network of lamellipodia to generate tension. Lamellipodia are typically mobile F-actin elements at the leading edge of the cell that are initiated by actin nucleation at the plasma membrane and generate treadmilling movements. Podosomes are more dynamic, consists of an actin core and a ring complex, and tend to display a gliding movement [4].

Podosomes are involved in important cellular processes such as extracellular matrix degradation, bone resorption by osteoclasts [3], and trans-cellular diapedesis of lymphocytes [5]. Besides adhesion and migration, podosomes enable DCs to degrade the connective tissue just behind the leading edge by using matrix metalloproteinases [6]. In DCs, podosome dynamics is also regulated by toll-like receptor (TLR) signaling. Upon activation by TLR ligands such as lipopolysaccharide (LPS) DCs transiently disassemble podosome structures *in vitro* accompanied by reduced migratory capacity and increased endocytosis. After approximately two hours DCs reassemble podosomes and resume migration [7], [8]. It has been proposed that this transient loss of migration prevents DCs from leaving the area of pathogen encounter and thus leads to enhanced local antigen uptake. The mechanisms with which DCs and other cell types regulate podosome formation, stability, disassembly and function, however, remain to be fully described. Podosomes consist of an F-actin-rich core that is surrounded by a ring or matrix of adhesion and scaffolding proteins, including integrins, paxillin, gelsolin, vinculin, and talin. Several proteins that are related to F-actin dynamics, such as ARP2/3, WASP, the small RhoGTPases Cdc42 and Rac1, and cofilin, have been shown to regulate the F-actin network within podosomes [3], [9].

Murine SWAP-70 regulates integrin-mediated adhesion and migration of B cells, mast cells, DCs, and erythroid cells [10]–[14]. In DCs, SWAP-70 localizes to the cytoplasm, and dependent on the activation status of the cells, may localize to cytoplasmic membranes, at sites of cell–cell contact, to micropinosomes, and co-localizes with RhoGTPases and F-actin [12], [15]. Functionally, SWAP-70 supports surface localization of peptide-loaded MHC class II [12] and regulates S1P induced motility of DCs [11]. SWAP-70 binds PIP_3_, F-actin, cofilin, and RhoGTPases [12], [14], [16], [17]. We demonstrated that SWAP-70 preferentially interacts with active RhoA and Rac1 in lysates of stimulated DCs, contributes to activation of Rac1 in DCs, and binds to GTP-loaded and non-loaded Rac1 or RhoA [11], [12], [14], illustrating the complex relationship between SWAP-70 and RhoGTPases. Unlike immature wild-type (wt) bone marrow-derived DCs, immature *Swap-70^–/–^* DCs contain constitutively active RhoA [12]. Recently, we have shown that SWAP-70 deficient osteoclasts are impaired in formation of F-actin rings, which are assembled from podosomes [18], [19], and in bone resorption [20]. Together with its role in regulation of the F-actin cytoskeleton these findings suggested a role of SWAP-70 in DC podosome formation and dynamics, which we set out to test in this study.

## Materials and Methods

### Mice

*Swap-70^–/–^* and isogenic wt mice of the 129/SvEMS strain were described before [21]. Animals were bred and maintained under pathogen-free conditions Experimental Center of the Medizinisch-Theoretisches Zentrum of the Medical Faculty at the Dresden University of Technology according to approved animal welfare guidelines, permission number 24-9168.11-1/2011-13 granted by the State of Saxony. The university animal welfare committee of the Medical Faculty Carl Gustav Carus, TU Dresden has approved this study, recommended approval to the State officials (who will grant the final permission), who then granted permission as described above.

### Cell culture

DCs were generated from mouse spleens as described elsewhere [7] with small modifications. Briefly, spleens from 6- to 8-week-old *Swap-70^+/+^* and *Swap-70^–/–^* mice were homogenized through a cell strainer. After red blood cell lysis, cells were washed once and seeded at a density of 1×10^6^ cells/ml in DMEM (Invitrogen) supplemented with 10% fetal calf serum (FCS) (Invitrogen), 1% Penicillin/Streptomycin (10000 U/ml/10 mg/ml, Biochrom AG), 50 µM 2-mercaptoethanol, 10% GM-CSF supernatant obtained from J558 cells, and 1 ng/ml recombinant human TGF-β (R&D Systems or PeproTech) into a 24 well plate (1 ml/well) at 37°C in a humidified atmosphere and 5% CO_2_. Every 4 to 5 days half of the medium was changed. DCs were used at days 12 to 16 of culture at which point 80 to 90% of the cells were CD11c positive as analyzed by FACS (data not shown).

### Immunocytochemistry

Podosome dynamics were analyzed by staining DCs for F-actin and vinculin and analyzed by confocal laser scanning microscopy. DCs were harvested and seeded on poly-L-lysine coated glass coverslips in a 24 well plate (2×10^5^ cells/well in supplemented medium) and incubated for different time periods at 37°C and 5% CO_2_. For some experiments DCs were incubated with 1 µg/ml LPS (Salmononella enterica, Sigma) for 60 min additionally. Coverslips were washed once with prewarmed PBS followed by fixation with 3.6% formaldehyde in PBS at room temperature (RT) for 10 min. After permeabilization using 0.1% Triton X-100 cells were stained with 13.2 nM rhodamine phalloidin (Invitrogen) and 6.5 µg/ml monoclonal anti-vinculin FITC conjugate (Sigma) in PBS at RT for 1 h. Stained cells were washed three times with PBS, mounted with Fluoromount-G (SouthernBiotech) containing 1 µg/ml DAPI (4′,6-diamidino-2-phenylindole), visualized using a Zeiss LSM 510 confocal laser scanning microscope, and processed with ImageJ software (http://imagej.nih.gov/ij/).

### DC retroviral transduction

To analyze podosome turnover, DCs were transduced with a vector coding for GFP-tagged actin using a retroviral transduction system as previously described [12] and analyzed with fluorescence activated cell sorting (FRAP). Retrovirus was produced by transfecting the Phoenix Eco 293T packaging cell line with a vector coding for actin-GFP. DCs were then infected with the retrovirus containing supernatant by spin infection at days 9 and 10 of culture. The plate was centrifuged for 60 to 90 min at 1000 x g at 37°C and subsequently incubated overnight at 37°C and 5% CO_2_. The retrovirus containing supernatant was changed the next morning approx. 14 h post-infection. Infection procedure was repeated once more as described. GFP expression was checked by fluorescence microscopy and DCs were analyzed at day 15 of culture using FRAP.

### FRAP analysis of podosome turnover

DCs expressing actin-GFP were harvested at day 15 of culture and seeded at 2×10^5^ cells per dish onto poly-L-lysine coated glass bottom microwell dishes (35 nm dish, 20 nm glass bottom, MatTek) and incubated overnight at 37°C and 5% CO_2_. FRAP experiments were performed using a Leica TCS SP5 confocal system (Leica) at 37°C without CO_2_ source. Experiments and data analysis were performed using the Leica FRAP application wizard, images were processed with ImageJ software. Each experiment was conducted at 100% laser power, acquiring 5 images with acousto optic tunable filter (AOTF) set to 30%, 10 bleaching images with AOTF set to 100% with zoom-in, and 70 images of fluorescence recovery with AOTF set to 30%. The time between frames was minimized. The fluorescence recovery half-time, t_1/2_, was calculated as the time necessary for the fluorescence signal to recover to 50% of the asymptote intensity.

### Matrix degradation

Glass coverslips were coated with Oregon Green 488-labeled gelatin (Invitrogen) (10 µg/ml) for 30 min at RT and subsequently stabilized with 3.6% formaldehyde in PBS for 15 min. After washing extensively with PBS and medium the coverslips were blocked with medium containing 10% FCS for 30 min. Coverslips were washed once more with medium and 2.5×10^6^ DCs per coverslip/well (24 well plate) were added in complete DC medium (500 µl/well). The cells were then incubated for 4 h before fixation with 3.6% formaldehyde, permeabilization with 0.1% Triton X-100, and staining with 13.2 nM rhodamine phalloidin (Invitrogen). Coverslips were mounted with Fluoromount-G (SouthernBiotech) containing 1 µg/ml DAPI and visualized using a Zeiss LSM 510 confocal laser scanning microscope. Matrix degradation was quantitated by measuring the area where the Oregon Green gelatin signal was below the threshold and counting the number of cells with ImageJ software (http://imagej.nih.gov/ij/).

### Statistical analysis

Statistical analysis and graphing were performed using Prism 5 (GraphPad) or Excel (Microsoft). Unpaired two-tailed student's t-test (confidence interval 95%) was employed to determine statistical significance. Error bars indicate standard deviation (SD). N.S. denotes not significant.

## Results

### SWAP-70 is present at podosome sites in DCs

SWAP-70 regulates RhoA activity in bone marrow-derived DCs and has been shown to stabilize actin filaments directly and indirectly thereby controlling F-actin dynamics [12], [22]. Moreover, we have demonstrated very recently that SWAP-70 deficient osteoclasts are impaired in formation of distinct podosome structures and bone resorption capacity [20], known to require RhoA activity for their formation [19]. Thus, an involvement of SWAP-70 in controlling DC podosome dynamics appeared very likely.

**Figure 1:**
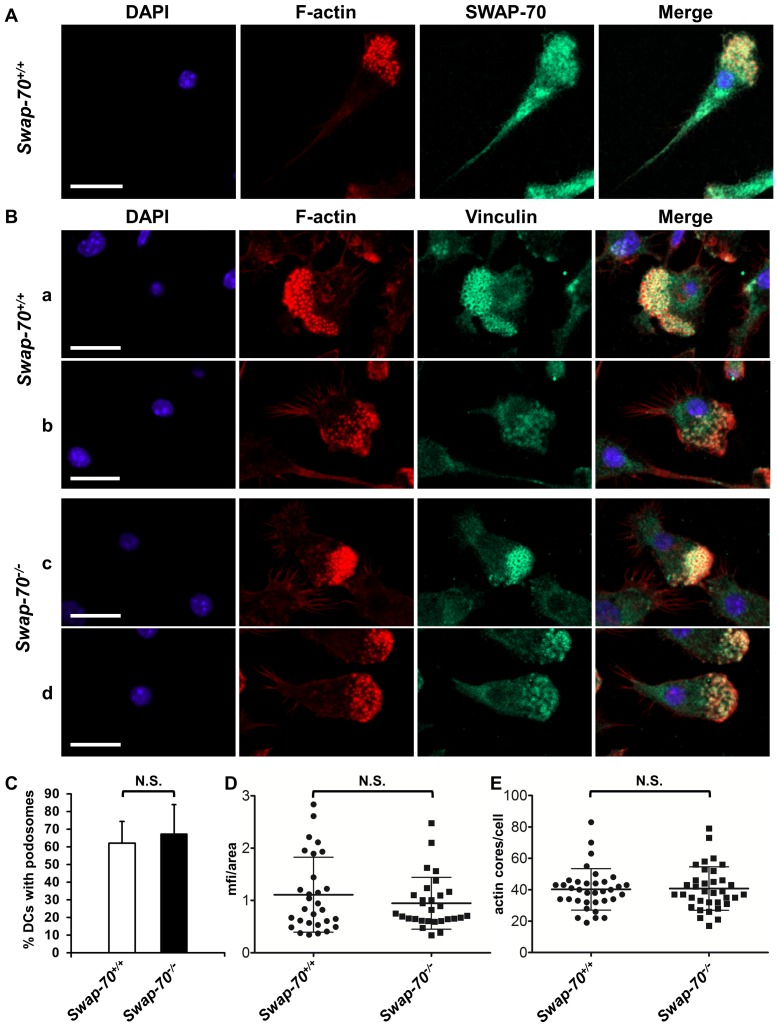
SWAP-70 localizes to podosome clusters but podosome formation is not affected by SWAP-70 deficiency in DCs. (**A**) *Swap-70^+/+^* DCs were stained with anti-SWAP-70 (green), rhodamine phalloidin for F-actin (red), and DAPI for the nucleus (blue) and analyzed with confocal laser scanning microscopy. Representative picture is shown. (**B**) Confocal immunofluorescence microscopy analysis of two individual *Swap-70^+/+^* (**a**, **b**) and *Swap-70^–/–^* (**c**, **d**) DCs. DCs were seeded and incubated for at least 2 h and stained with anti-Vinculin (green), rhodamine phalloidin for F-actin (red), and DAPI for the nucleus (blue). (**C**) Quantification of the number of DCs exhibiting podosome clusters. >100 cells were analyzed per genotype and experiment. Mean values of 4 independent experiments with error bars indicating +SD are shown. (**D**) Quantification of the mean fluorescence intensity (mfi) per area of podosome structures by measuring fluorescence intensity of rhodamine phalloidin staining (F-actin). Mean values of 30 individual cells per genotype of 2 independent experiments with error bars indicating ±SD are shown. (**E**) Quantification of the number of podosome actin cores per cell. Mean values of 35 individual cells per genotype of 2 independent experiments with error bars indicating ±SD are shown. N.S. denotes not significant. Scale bars, 20 µm.

To test this hypothesis, *Swap-70^–/–^* DCs were analyzed. SWAP-70 is mainly found in the cytoplasm in steady state situations but can localize to the membrane or the nucleus upon stimulation [15], [23]. Interestingly, our group observed recently that SWAP-70 accumulates in close proximity to the podosome belt in mature osteoclasts [20]. To investigate SWAP-70 localization in immature podosome bearing DCs, wt DCs differentiated from splenocytes were co-stained with phalloidin for F-actin and SWAP-70 and analyzed using confocal microscopy. SWAP-70 was shown to be present at sites of podosome structures characterized by the typical actin core and ring structure at the leading edge but does not specifically localize to the protein cloud surrounding the F-actin core in spleen-derived DCs (Figure 1A) and bone marrow-derived DCs (data not shown).

### Podosome formation is independent of SWAP-70 in DCs

To address whether SWAP-70 plays a role in proper formation and structure of podosomes in DCs, podosomes were analyzed by staining DCs of wt and *Swap-70^–/–^* mice with phalloidin for F-actin and an antibody for the podosome-associated protein vinculin (Figure 1B). DCs of both wt and *Swap-70^–/–^* mice showed the characteristic podosome pattern with a dense phalloidin stained F-actin core (red) surrounded by vinculin (green) staining located typically at the leading edge of cells (Figure 1B, a-d) [24]. F-actin cores varied between each wt or *Swap-70^–/–^* DC, presumably representing different stages of podosome formation. One representative cell of more dense (a, c) and more distant actin core (b, c) podosomes of each wt (a, b) and SWAP-70 deficient (b, c) DCs are shown in figure 1B. The percentage of cells displaying podosome structures was quantified and no difference between wt and SWAP-70 deficient DCs was found (Figure 1C). Generally 50 to 85% of DCs exhibited vinculin-positive podosome structures. To investigate whether the F-actin density in podosome structures was affected by SWAP-70 deficiency, fluorescence intensity of phalloidin staining in podosome areas was analyzed. Mean fluorescence intensity (MFI) per area was measured and quantified showing no influence of SWAP-70 deficiency on F-actin density in DCs (Figure 1D). Additionally, the number of actin cores per cell within podosome structures was quantified. Although the number generally varied greatly (20 to 90 cores per cell), SWAP-70 deficiency did not affect the number of actin cores per cell affirming the above results (Figure 1E). Podosome formation in BMDCs was not affected by SWAP-70 deficiency either (data not shown). We thus conclude that podosome formation is independent of SWAP-70 in murine DCs.

### TLR-mediated loss of podosomes is independent of SWAP-70

**Figure 2:**
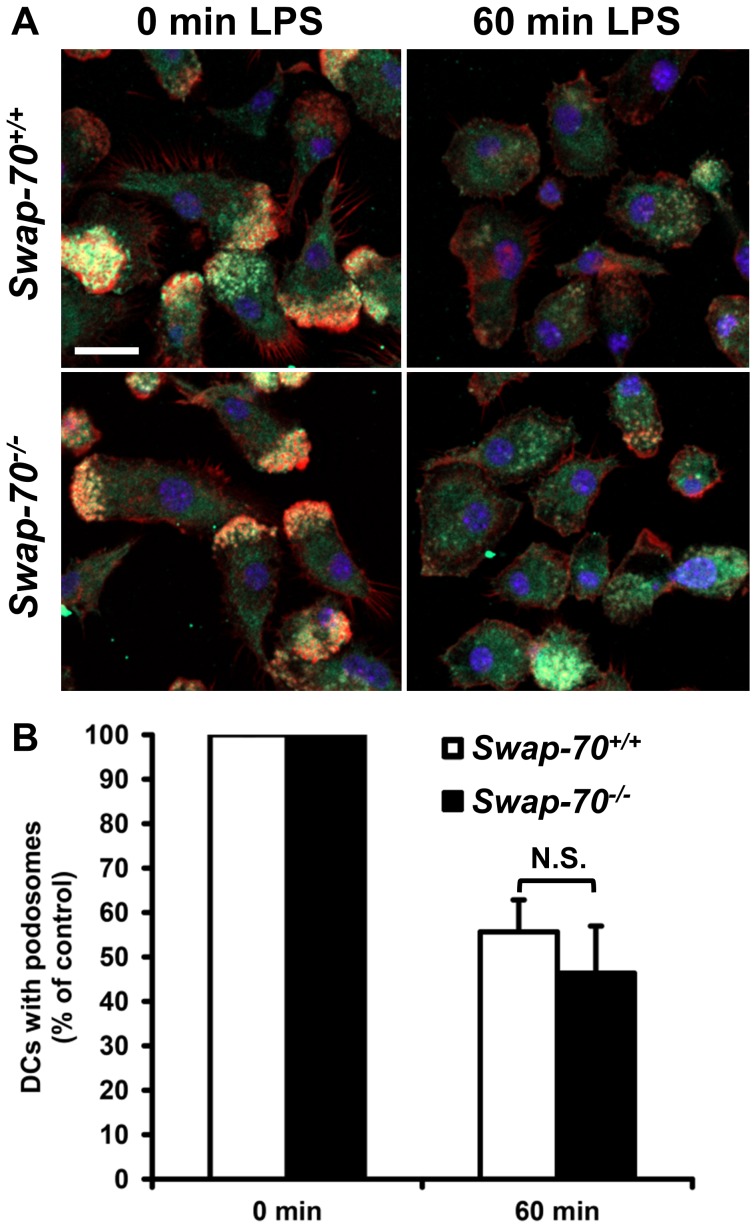
LPS-induced podosome disassembly is not affected by SWAP-70 deficiency in DCs. (**A**) *Swap-70^+/+^* and *Swap-70^–/–^* DCs were treated with or without LPS for 60 min. Cells were stained with anti-Vinculin FITC (green), rhodamine phalloidin for F-actin (red), and DAPI for the nucleus (blue) and analyzed by confocal laser scanning microscopy. (**B**) Quantification of the number of DCs exhibiting podosome clusters with and without LPS treatment for 60 min. Graph indicates the number of cells with podosomes after LPS treatment relative to control (without LPS treatment). Mean values of 3 independent experiments with error bars indicating +SD are shown. N.S. denotes not significant.

Next, we analyzed the dynamics of podosome assembly and disassembly in SWAP-70 deficient DCs. It has been shown previously that DCs start to disassemble podosome structures upon TLR-mediated activation within approximately 10 to 20 min after LPS addition [7]. To investigate if TLR-induced disassembly of podosomes requires SWAP-70, the percentage of wt and SWAP-70 deficient DCs after 60 min of LPS incubation was quantified and compared (Figure 2). After 60 min of TLR4-induced activation both wt and *Swap-70^–/–^* DCs showed significantly less cells exhibiting podosome structures (Figure 2A-B). However, we did not observe a significant difference in TLR-induced podosome disassembly between wt and SWAP-70 deficient DCs (Figure 2B). Quantification of DCs showing podosomes with and without LPS treatment revealed that approximately half of the cells had disassembled podosome structures after 60 min (Figure 2B). Analysis of earlier and later time points after LPS addition did not reveal differences either (data not shown). Hence, TLR-mediated decrease of podosome structures in DCs is independent of SWAP-70 as well.

### Podosome turnover is not affected by SWAP-70 deficiency

**Figure 3:**
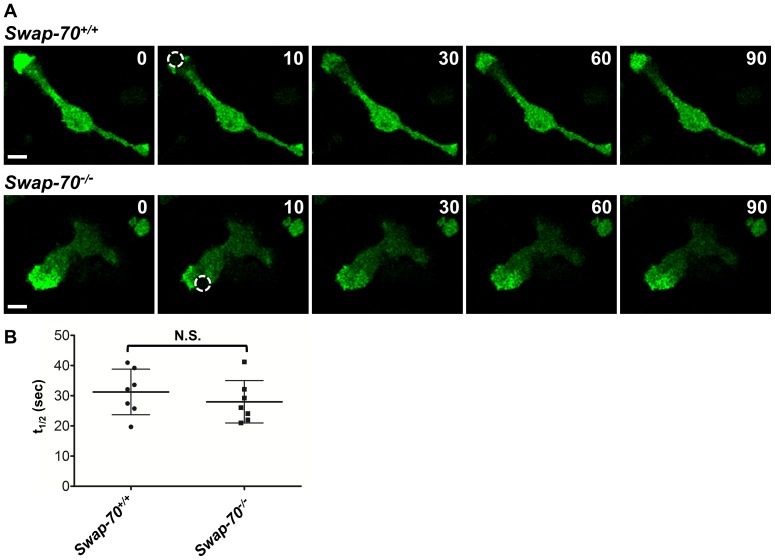
Podosome turnover is not affected by SWAP-70 deficiency in DCs. (**A**) FRAP analysis. Actin-GFP transduced *Swap-70^+/+^* and *Swap-70^–/–^* DCs were seeded on poly-L-lysine coated glass coverslips and discrete areas (white circles) were photobleached. Representative selected pictures are shown with times in seconds. (**B**) Quantification of fluorescence recovery half-time (t_1/2_) in seconds from experiments in (A). Mean values of 7 individual photobleached cells per genotype of 2 independent experiments with error bars indicating ±SD are shown. N.S. denotes not significant.

Wt and *Swap-70^–/–^* DCs were transduced with a retroviral vector coding for actin-GFP to analyze F-actin turnover in podosomes using FRAP. Transduced DCs displayed the typical actin-GFP dense podosome core clusters at the leading edge. After photo-bleaching of discrete circular areas, podosomes rapidly reincorporated actin-GFP as previously shown by others (West et al., 2004). In both wt and SWAP-70 deficient DCs, fluorescence of actin-GFP in podosomes reappeared after 30 to 90 sec after bleaching (Figure 3A). The fluorescence recovery in podosome cores fitted closely an exponential law with a characteristic dynamical time, t_1/2_, ranging from 20 to 40 sec, depending on the cell considered (Figure 3B) confirming published results in RAW267 cell-derived osteoclasts [25]. t_1/2_ represents the time required for half of the fluorescence intensity of actin-GFP to recover and as such can be used to compare actin turnover in individual cells. Quantification of t_1/2_ of FRAP experiments with wt and SWAP-70 deficient DCs revealed no significant difference in podosome turnover (Figure 3B).

### Matrix degradation capacity of DCs is not altered in the absence of SWAP-70

**Figure 4:**
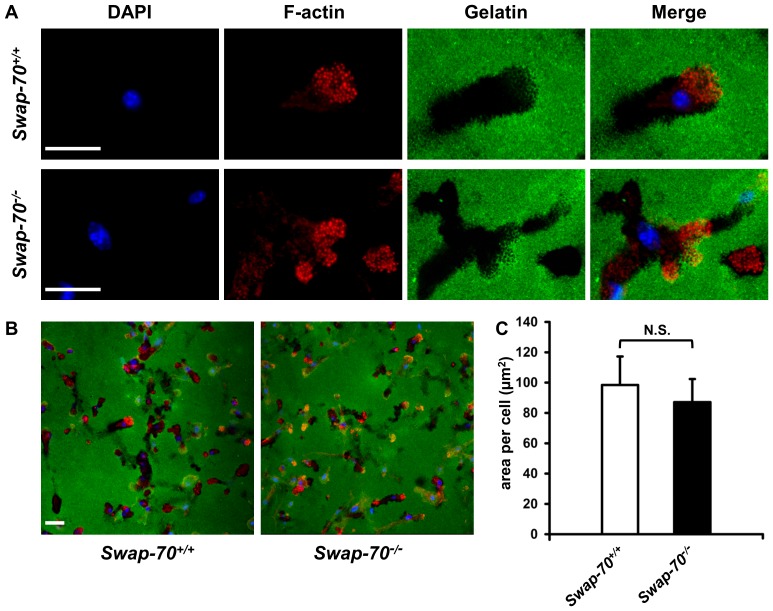
Podosome-mediated degradation of gelatin is not affected by SWAP-70 deficiency in DCs. (**A-B**) Confocal immunofluorescence microscopy analysis of gelatin degradation by *Swap-70^+/+^* and *Swap-70^–/–^* DCs. DCs were seeded on Oregon Green-labeled gelatin (green) and incubated for 4 h. Cells were stained with rhodamine phalloidin for F-actin (red) and DAPI for the nucleus (blue). Representative detailed (**A**) and overview (**B**) pictures are shown. (**C**) Quantification of the area (in µm^2^) of Oregon Green-labeled gelatin degraded by DCs. Mean values of 3 independent experiments with error bars indicating +SD are shown (300-500 cells were analyzed per genotype and experiment). N.S. denotes not significant.

*Swap-70^–/–^* osteoclasts are impaired in matrix degradation capacity and F-actin-ring formation [20]. To investigate whether DC podosomes of SWAP-70 deficient mice were similarly impaired in function, we examined their capacity to degrade extracellular matrix. DCs were plated on Oregon Green-labeled gelatin coated coverslips for 4 h and stained for F-actin before analysis using confocal laser scanning microscopy. Discrete holes in the gelatin matrix could be observed in the vicinity of podosome clusters (Figure 4A and B) as it has been shown before [8]. Additionally, this assay showed that podosome formation and occurrence was comparable in wt and *Swap-70^–/–^* DCs on a more natural substrate than poly-L-lysine. Quantitative analysis of the total area of degraded gelatin per cell revealed that there was no major difference between wt and *Swap-70^–/–^* DC's capacity to degrade matrix *in vitro* (Figure 4C). Thus, podosomes in SWAP-70 deficient DCs are functional in matrix degradation.

Taken together these results suggest that SWAP-70 is dispensable for podosome formation, F-actin turnover, LPS-induced disassembly, and matrix degradation function in murine DCs.

## Discussion

Podosomes are F-actin-based protrusions of the membrane that form close contact with the surrounding substrate and can be found in a wide variety of cells including endothelial cells, osteoclasts, macrophages, and DCs [9]. They are involved in migration, extracellular matrix degradation [6], and osteoclast bone resorption [3]. The importance of podosomes in cellular and systematic functions has been emphasized recently by the first report of podosome structures *in vivo* [26]. Among many other adhesion and scaffolding proteins, the characteristic actin-rich core of podosomes featuring proteins like gelsolin and cortactin, is surrounded by a cloud including integrins, vinculin, paxillin, cofilin, the RhoGTPases RhoA, Rac1, and Cdc42, and other proteins [3], [9], [18].

SWAP-70 controls RhoA, Rac1, and integrin activity [10], [12]–[14], [27], [28] and binds F-actin and cofilin [16], [29] (Chacón-Martínez et al., submitted). Additionally, we lately found SWAP-70 to bind to Cdc42 *in vitro* (unpublished data). Recently, we showed that SWAP-70 deficient osteoclasts exhibit impaired podosome-ring formation and bone resorption capacity [20]. In DCs, SWAP-70 deficiency leads to a delayed entry into lymph nodes upon activation and a decreased number of DCs present in the lymph nodes at steady state *in vivo* [11]. Although this decreased migration could be explained by SWAP-70's regulation of S1P signaling, impaired podosome regulation in SWAP-70 deficient DCs may contribute as well. Indeed, SWAP-70 localizes to podosomes in DCs.

However, the data reported here show that at least under the conditions applied in this study, SWAP-70 is not relevant for podosomes. In particular, SWAP-70 is not required for formation of podosomes in wt-like numbers per cell, for generating of normal-sized podosomes, for F-actin turnover within podosomes, for the loss of podosomes in LPS-activated DCs, and for the capacity of DCs to degrade matrix degradation. Hence, SWAP-70 seems to be dispensable, at least in the context of podosomes, for expression and secretion of matrix metalloproteases as well.

These findings indicate that SWAP-70 plays a role in podosome biology and biochemistry in osteoclasts but not in DCs. An explanation for this difference could be that SWAP-70 exhibits different modes of action in different cells types. SWAP-70 regulates adhesion in B cells, mast cells, and erythroid cells [13]. Moreover, a crucial role of SWAP-70 in B cells depends on its nuclear function to regulate class switching [21], [30] and plasma cell development [31], whereas in other cell types SWAP-70 is apparently exclusively acting in the cytoplasm or at the membrane. Another possible explanation is that differential expression of other proteins in DCs and osteoclasts compensate for the lack of SWAP-70 in DCs. For example, DEF6 (also called SLAT or IBP), the only protein closely related to SWAP-70 [32]–[34], may complement effects of SWAP-70 deficiency on podosomes in DCs. However, initial experiments using DCs deficient in either one or both proteins, SWAP-70 and DEF6, suggest that the lack of DEF6 does not affect podosome formation either (data not shown).

Another reason for the osteoclast-DC difference may be that podosomes in DCs and in osteoclasts are rather different structures with similar components but different fate. In osteoclasts, but not in DCs, the supra-molecular organization of the individual podosomes into podosome/F-actin rings, which then generate the sealing zone to form the resorption pit, may require SWAP-70. No such rings are formed by DCs. SWAP-70 localizes in close proximity to the inner side of the F-actin-ring in osteoclasts [20]. Thus, SWAP-70 might only regulate the formation of larger F-actin rings rather than affecting the individual podosome cores. Similar to SWAP-70, the actin severing protein gelsolin regulates podosome formation and function in osteoclasts [35] but not in DCs [36]. It seems logical that podosomes of different cell types that have distinct functions exhibit also differences in their regulation. Future investigations have to focus on distinct requirements of specific cell types.
